# Annotations

**Published:** 1891-02-07

**Authors:** 


### Annotations.

**The Nurses' Co-operation.**

The Press, which in spite of its ten thousand mistakes and extravagances, has an excellent eye for what is plainly and obviously good, has decided to bestow upon the Nurses' Co-operation a universal benediction. If merit alone can make an enterprise succeed, the Co-operation can by no possibility fail. It is based upon a principle of justice of the most elementary and manifest kind. That a man should have the fullest right to every penny which he himself honestly earns, is a proposition which neither the political economist nor the moral philosopher will for a single moment doubt. But if this be true of so sturdy and self-reliant a creature as man, much more is it true of a shrinking and timid woman. We are not among those who have anything to say against the supplying of trained nurses to the public by hospitals or by private agencies. On the contrary, we do not doubt that the hospitals and the private agencies have done, and are doing, both a good and a necessary work. Nurses can no more be brought with promptitude to the sick beds of private patients without central agencies than the messages of the telephone can be distributed to the various parts of the country without a telephonic centre. When the public first began to know the value of hospital trained nurses, nothing was more natural than that they should apply to hospitals for them, and that the hospitals should respond to the application. As the demand for the services of nurses increased, and as the difficulty also increased of obtaining those services promptly in every part of the country, it was quite in accordance with the laws of natural development that enterprising private persons should see profit in acting the part of middlemen and bringing together easily the nurse and the patient who were often placed by circumstances so very far asunder. Just as the two former methods have been natural developments, so is the Nurses' Co-operation a still further development on the same lines. Nurses of independent minds will naturally join the Co-operation, and take the risks and the profits. Others who are less stout of heart will seek protection under the shadow of the hospital and the private agency. There is room for all these methods of distribution; and they may all do good work. We hope they will not be so senseless as to quarrel. The best course for the hospitals and the private agencies to pursue is to enter into combination with the new co-operation. All women workers should be on one side, and especially all nurses. When workers quarrel among themselves it is the common adversary who reaps the profit. Universal co-operation is the true way of safety.

**One-Roomed Life in London.**

The Rev. J. Munro selected a title for his lecture at the Richmond Athenæum last Monday, which had the merit of a little novelty. "Life in London" has not only been acted upon the stage, but has probably served as a heading for many sermons, and as the title of at least one book. "One-Roomed Life" was probably used for the first time when Mr. Munro employed it. We do not know what the lecturer actually said to his audience, but we do know what he might have said. One-roomed life, if carried out extensively enough, is sufficient of itself, not only to ruin London, but also to wreck the Empire, and to destroy the British character. The working man, like all other men, consists of body and mind. In one room neither body nor mind can be well—they must both be sick, and mortally sick. Illness of body always causes death if it continue long enough, and so does illness of mind. Probably every reader will consider that our propositions require no proof. That we admit; but they require something much more difficult than proof—they demand action. When a farmer and his wife ride in one gig to market they can travel with great comfort; but when on some special occasion, say a cricket match or an agricultural show, not only the farmer and his wife, but also his two sons and his three daughters have to go to the market town, and the gig is the only available vehicle, then, indeed, things assume a very different complexion. The farmer must think, and so must his wife, and so must his sons and his daughters; and they must think to some purpose, or they will never get to the cricket match in that particular gig. They may not all get there in one, and possibly not in two journeys, even if they do think. But they certainly will never get there at all if they do not think. Two or three hundred years ago, when the population of London was small, and space was abundant, there was no necessity for whole families of people to live day and night in one and the same room. The need for thinking about the subject had not arisen, any more than it arises when the farmer and his wife set out by themselves in their gig on an ordinary market day. But now the necessity has arisen; and it is a dire, and may prove a fatal, necessity. The stuffing of whole families into one room is sad, bad, and mad. We do not know how to stigmatise it sufficiently. The governing classes are responsible for it, and ought to be ashamed of it. We do not mean that they should be ashamed of their wickedness, but of their stupidity and incompetence in permitting the thing to reach such a pitch as it has reached. Somebody ought to mend matters! But who? Nobody can mend them except the governing classes. They pride themselves on being the governing classes; then let them so govern that at least the main elements of public well-being shall be conserved. What are those main elements? They are bodily health and decency of behaviour. Neither of these is possible to families who live in one room. The British are a great people; but the greatest of peoples can be destroyed if their environment be sufficiently injurious. Our rulers have the power and the means of furnishing their less fortunate fellow countrymen with a perfectly healthy environment. If they do not do this their want of courage and ruling capacity will wreck the greatest empire and degrade to ruin the noblest people that the world has ever seen.

**Anti-Vaccinators at Tunbridge Wells.**

Laws which are not obeyed had perhaps better be abolished; but until they are abrogated it seems necessary to put them in force. At the same time, as orthodox believers in the prophylactic value of vaccination, we cannot regard with satisfaction the scenes which were witnessed at Tunbridge Wells a week or two ago. The goods of a Mr. Ingram were seized under a distress warrant in pursuance of the Vaccination Acts, and a public sale of them was announced. The fine was apparently very small, and a pair of counter scales seems to have been the only seizure. A large crowd assembled at the sale, whose chief business appears to have been to chaff and banter the auctioneer. Subsequently an indignation meeting, presided over by Alderman Clifford, was held, and the anti-vaccinators were received by the audience with great demonstrations of enthusiasm. Mr. Ingram, in the course of his speech, referred to the case of Mr. Mitchell, who was distrained on under the Acts whilst his child was lying dead, and his wife dying in the house. Under these circumstances the Anti-Vaccination Society paid the fine. It is time for medical men to ask themselves the question whether it is worth their while to continue to incur the odium of supporting compulsory vaccination? We can all continue to believe in vaccination itself, and we can continue to vaccinate our own children, and the children of those of our patients who have faith in vaccination, and in us. But it is clear that whoever gains by compulsory vaccinal prophylaxy the doctor does not; and what is the use of his forcing measures of safety upon an ungrateful public, when his advocacy of such measures results to himself in nothing but a monetary loss and personal odium? We believe in vaccination; and so long as we continue to believe in it we shall practise it for our own safety and that of our friends. But if a section of the public chooses to doubt our prophylactic methods, let it take the risk upon itself. We may be kind, but we are certainly not wise in holding up a torch in the darkness when so many are bent only upon snatching the torch from our hands, and pushing it, all ablaze, into our faces. There is high authority against casting our pearls before swine.

